# Efficient Gene Expression in Human Stem Cell Derived-Cortical Organoids Using Adeno Associated Virus

**DOI:** 10.3390/cells11203194

**Published:** 2022-10-11

**Authors:** Ann-Na Cho, Fiona Bright, Nicolle Morey, Carol Au, Lars M. Ittner, Yazi D. Ke

**Affiliations:** Dementia Research Centre, Macquarie Medical School, Faculty of Medicine Health and Human Sciences, Macquarie University, Sydney, NSW 2109, Australia

**Keywords:** cortical organoids, Adeno-associated viruses (AAVs), TDP-43, gene delivery, neurodegenerative disease

## Abstract

Cortical organoids are 3D structures derived either from human embryonic stem cells or human induced pluripotent stem cells with their use exploding in recent years due to their ability to better recapitulate the human brain in vivo in respect to organization; differentiation; and polarity. Adeno-associated viruses (AAVs) have emerged in recent years as the vectors of choice for CNS-targeted gene therapy. Here; we compare the use of AAVs as a mode of gene expression in cortical organoids; over traditional methods such as lipofectamine and electroporation and demonstrate its ease-of-use in generating quick disease models through expression of different variants of the central gene—TDP-43—implicated in amyotrophic lateral sclerosis and frontotemporal dementia.

## 1. Introduction

The three-dimensional, self-organizing structure of brain organoids derived from human pluripotent stem cells (hPSCs) enables recapitulation of the cellular composition and functionality of the human brain and provides insights into its physiological and pathological processes leading to brain diseases [1,2,3,4,5]. Brain organoids are of particular interest in the context of aging, neurodegenerative disease, and other neurological disorders given their ability to spatially model complex interactions between diverse cell-types in vitro, approximating to the human in vivo brain context [6,7,8,9,10,11,12,13,14,15]. Furthermore, brain organoids can be utilised to explore and understand the assembly of neural circuits and cell–cell interactions in vivo, by manipulating such interactions and processes in real time [16,17,18,19,20]. Human cortical organoids (hCOs), also known as cortical spheroids, and cerebral organoids are examples of brain organoids with varying degree of complexity in terms of cellular organization and composition due to the presence of exogenous patterning factors. Here we adopted the protocol developed in the lab of Sergiu Pasca, growing hCOs containing deep and superficial cortical neurons as well as quiescent astrocytes [1].

Adeno-associated viruses (AAVs) have emerged in recent years as the vectors of choice for CNS-targeted gene therapy to study disease mechanisms in animal models as well as therapeutic options in neurological diseases [21,22,23]. The popularity of AAV for CNS gene delivery can be explained by their non-pathogenic and replication-deficient nature, their ability to transduce neurons as well as their ability to convey long-term transgene expression with minimal immune response. The wild-type AAV genome can be turned into a recombinant AAV vector genome by replacing the rep and cap genes with an expression cassette of choice consisting of a promoter that is active in the target tissue, the gene of interest, and a polyadenylation signal [21,23]. This ease-of-use of AAVs can be harnessed to express many different gene variants at the same time for effective study of target genes identified in familial diseases such as amyotrophic lateral sclerosis (ALS) and frontotemporal dementia (FTD) [24,25,26].

Here, we performed a comparative study of gene delivery methods to assess gene delivery efficiency in hCOs using adeno-associated viruses (AAV) compared to lipofectamine and electroporation. Next, we used AAV to express the ALS/FTD pathological protein, human TDP-43 to demonstrate that disease-like features can be achieved in hCOs using this gene delivery protocol [27,28,29,30,31,32,33,34].

## 2. Materials and Methods

### 2.1. Human Embryonic Stem Cells (hESCs) Maintenance and Generation of hESCs-Derived Cortical Organoids (hCOs)

The hESC-derived cortical organoids were produced as previously reported [1]. Briefly, the hESCs (H9; WIC-WA09-MB-001, WiCell, Madison, Wisconsin) were maintained on 6-well plates coated with Matrigel (#354230; Corning, Corning, NY, USA) in mTeSR 1 medium (#85850; STEMCELL Technologies, Vancouver, BC, Canada) at 37 °C, 5% CO_2_. When hESCs reached 80–90% confluency, cells were rinsed with DPBS (#14190144; Thermo Fisher Scientific, Waltham, MA, USA) prior to addition of ReLeSR (#05872; STEMCELL Technologies). Cells were incubated for 7 min at 37 °C, resuspended in mTeSR and passaged onto freshly prepared Matrigel-coated 6-well plates. To generate hCOs, hESCs were treated with StemPro Accutase Cell Dissociation Reagent (#A1110501; Thermofisher scientific) at 37 °C for 1 min. The dissociated single cells were seeded on AggreWell^TM^ 800 plates (#34815; STEMCELL Technologies) containing 1200 microwells. 1000 single cells were added per each well in AggreWell EB Formation Medium (#05893; STEMCELL Technologies) supplemented with the ROCK inhibitor Y-27632 (10 μM, #72308; STEMCELL Technologies), and centrifuged STEMCELL Technologiesat 100× *g* for 3 min. The cells were incubated at 37 °C with 5% CO_2_. After 24 h, embryoid bodies were collected from each microwell by pipetting, then transferred into ultra-low-attachment 10 cm dishes (#CLS3262; Corning) in Essential 6 medium (#A1516401; Thermo Fisher Scientific) supplemented with dorsomorphin (2.5 μM, #P5499; Sigma-Aldrich, St. Louis, MO, USA), SB-431542 (10 μM, #1614; Tocris, Bristol, UK) and XAV-939 (2.5 μM; #3748, Tocris). Medium was changed every day for 5 days. At day 6, medium was change into Neurobasal A (#10888; Thermo Fisher Scientific) containing 1X B-27 supplement without vitamin A (#12587; Thermo Fisher Scientific) and 1X GlutaMax (#35050; Thermo Fisher Scientific). The medium was supplemented with 20 ng/mL epidermal growth factor (EGF, #236-EG; R&D Systems, Minneapolis, MN, USA) and 20 ng/mL basic fibroblast growth factor (bFGF, # 233-FB; R&D Systems) until day 24. At day 24, medium with Neurobasal A (Thermo Fisher Scientific), 1X B-27 supplement without vitamin A (Thermo Fisher Scientific) and 1X GlutaMax (Thermo Fisher Scientific) was instead supplemented with 20 ng/mL brain-derived neurotrophic factor (BDNF, #78133; STEMCELL Technologies) and 20 ng/mL Neurotrophin-3 (NT3, #78074; STEMCELL Technologies) until day 43. Organoids were maintained in Neurobasal A (Thermo Fisher Scientific), 1X B-27 supplement without vitamin A (Thermo Fisher Scientific) and 1X GlutaMax (Thermo Fisher Scientific) without growth factors from day 43. For organoid analysis, at least 4 organoids were taken from each batch and timepoint for characterization studies.

### 2.2. Immunofluorescence Staining

For the whole-mount staining of 30-day old organoids, the hCOs were fixed in 4% PFA for 30 min at room temperature, washed three times using PBS, and permeabilized with 0.5% (*v*/*v*) Triton X-100 (#X100; Sigma-Aldrich) diluted in PBS for 30 min. The hCOs were subsequently incubated in 5% (*w*/*v*) bovine serum albumin (#A7030; Sigma-Aldrich) for 5 h to block non-specific binding of antibodies and further incubated with primary antibodies listed in Supplementary Table S1 at 4 °C for 3 days. The organoids were washed with PBS three times and incubated with Alexa Fluor-conjugated secondary antibodies (Thermo Fisher Scientific) at 4 °C for 3 days and counterstained with 4′,6-diamidino-2-phenylindole (DAPI, #D1306; Invitrogen, Waltham, MA, USA) for 30 min and washed with PBS.

Day 40, 50, and 100—old hCOs were embedded in paraffin and were sectioned at 3µm thickness with a Thermo HM325 microtome. The sections were deparaffinated, rehydrated, and blocked, followed by overnight incubation at 4 °C with primary antibodies as listed in Supplementary Table S1. The sections were washed with PBS two times and incubated with Alexa Fluor-conjugated secondary antibodies (Thermo Fisher Scientific) at room temperature for 1 h. The nuclei were counterstained with DAPI for 10 min and mounted in Vectashield Plus antifade mounting medium (#H-1000; Vector Laboratories, Newark, CA, USA). Immunofluorescence imaging was carried out on a confocal microscope (LSM 880; Carl Zeiss, North Ryde, Australia).

### 2.3. Quantitative Real-Time PCR

The gene expression of the hCOs at each time point (day 30, 40, 50, 60, 70, and 100) was investigated by quantitative real-time PCR (qPCR) analysis. The organoids were further purified using RNeasy Mini Kit (#74104; Qiagen, Hilden, Germany) following the manufacturer’s instructions. Complementary DNA was synthesized from 1µg RNA using SuperScript VILO cDNA synthesis kit (#11754050; Thermo Fisher Scientific). Messenger RNA levels were determined by qPCR using Power SYBR Green Master Mix (#4367659; Thermo Fisher Scientific) and gene-specific primer pairs listed in Supplementary Table S2, using an Applied Biosystems ViiA 7 Real-Time PCR System and QuantStudio Real-Time PCR software (Themo Fisher Scientific). The relative gene expression level of each primer was calculated by the comparative Ct method and normalized to endogenous reference glyceraldehyde 3-phosphate dehydrogenase (GAPDH). All primers used are listed in Supplementary Table S2.

### 2.4. Lipofectamine Transfection

The day 30-old hCOs were prepared for Lipofectamine transfection using three different types of Lipofectamine (2000 Transfection Reagent; #11668019, Stem Transfection Reagent; #STEM00001, 3000 Transfection Reagent; #L3000001). Amounts of 1.6μg of plasmid DNA for EGFP and 1μL of Lipofectamine reagent per organoid were prepared following the manufacturer’s instructions. Organoids were incubated in this DNA/lipofectamine mixture. The medium was replaced after 72 h treatment and organoids were analysed at day 7 under the confocal microscope.

### 2.5. Electroporation and BrainFectIN

Day 30 hCOs were electroporated using the Neon Transfection System (#MPK1025; ThermoFisher Scientific) following the manufacturer’s instructions. In brief, the organoids were washed twice in DPBS with no calcium, no magnesium (#14190144; Thermofisher Scientific) and resuspended in buffer R containing 0.4 and 1 µg of the plasmid DNA. The electroporation conditions for hCOs were voltage 500 V, width 50 ms, and 5 pulses. The electroporated organoids were cultured in fresh medium and analyzed at day 7.

The hCOs were transfected by BrainFectIN (#IV-BF30500; OZ Biosciences, San Diego, CA, USA) as manufacturer’s instructions. Briefly, 3 µL of BrainFectIN was mixed with 2 µg of plasmid DNA and directly applied into the medium of day 30 organoid. The fresh medium was replaced at 3 days and fluorescence expression was investigated after 7 days.

### 2.6. AAV Production and Transduction

The AAV preparations of the following plasmids: AAV hSyn TDP-43 WT-myc, AAV hSyn TDP-43 A315T-myc, AAV U6-shRNA-scramble-GFP (VectorBuilder, Chicago, IL, USA), pGP-AAV-syn-jGCaMP7f-WPRE (gift from Douglas Kim & GENIE Project; Addgene plasmid # 104488) AAV hSyn-eGFP (gift from Bryan Roth; Addgene plasmid # 50465;) were carried out as previously described [35]. Briefly, 293T cells were seeded in complete DMEM (Sigma) with 10% FBS and medium was changed to IMDM (Sigma) with 5% FBS 3 h prior to transfection. Cells were transfected with AAV vector of interest, pFdelta6 as helper plasmid and AAV-PHP.B plasmid containing rep and cap sequences using polyethyleneimine-Max (PEI-Max, Polysciences, Warrington, PA, USA). Cells and supernatant were harvested 72 h post transfection and clarified in 40% PEG8000/2.5 M NaCl to a final concentration of 8% PEG8000/0.5 M NaCl at 4 °C for 2 h and centrifuged at 2000× *g* for 30 min. Combined precipitate was subsequently treated with sodium deoxycholate (0.5% final concentration) and benzonase (~500 U) at 37 °C for 40 min. After addition of NaCl, incubation at 56 °C for 40 min and freeze–thaw, the solution was centrifuged 30 min at 5000× *g* at 4 °C. Supernatants were purified using iodixanol gradient by ultracentrifugation (475,900× *g* for 2 h at 18 °C). AAV particles were concentrated in an Amicon Ultra-15 100 kDa concentrator at 4000× *g* at 4 °C. Titres were determined by qPCR. Viral transduction of organoids was performed in Ultra-low attachment 96 well-plate (#CLS3474; Corning) for 3 days. After a 3-day infection, fresh medium was added.

### 2.7. RIPA Extraction and Western Blotting

The hCOs were homogenized and sonicated in RIPA buffer (50 mM tris (pH 8), 150 mM NaCl, 0.1% Na-dodecyl sulphate, 0.5% Na-deoxycholate, 1% Nonidet™ P 40 substitute, 5 mM EDTA and protease inhibitor cocktail (cOmplete™, Roche)). Lysates were then centrifuged at 14,000 rpm at 4 °C and supernatant was collected for protein measurements. Western blotting was carried out as previously described [36] and probed for the corresponding tags of the AAV used (myc or eGFP; Supplementary Table S2). GAPDH was used for normalization.

### 2.8. Calcium Imaging

Day 50 hCOs were treated with AAV-PHP.B-hSyn-jGCaMP7f-WPRE and fresh medium was changed after a 3-day treatment. Organoids were cultured for an additional 14 days, then 64-day old organoids were analysed under confocal microscope. For the live imaging, organoids were incubated in artificial cerebrospinal fluid (ACSF) buffer (mmol/L: 26 NaHCO_3_, 10 dextrose, 1 MgSO_4_·7H_2_O, 1.25 NaH_2_PO_4_, 2.5 KCl, 126 NaCl, 2 CaCl_2_), pH 7.3 [37]. For a functionality check, 100 nM glutamate was used to evoke calcium influx, 25 uM gabazine was applied to block GABA_A_ receptor activity, and 300 nM tetrodotoxin (TTX) was used for sodium channel blocking. The live imaging was carried out on a confocal microscope (LSM 880, Zeiss, Jena, Germany) using 10×objective and the ROIs were measured using the ZEN software and data analysed using GraphPad Prism 9.0.

### 2.9. Statistical Analysis

All data are displayed as mean ± SD. 4 replicates per each sample was investigated. Statistical differences between two groups were tested by two-tailed unpaired Student’s *t* tests. All graphical data are presented by GraphPad Prism 9.0. A *p*-value below 0.05 was accepted as significant.

## 3. Results

### 3.1. Reliable Production of hESCs-Derived Cortical Organoids (hCOs)

**Figure 1:**
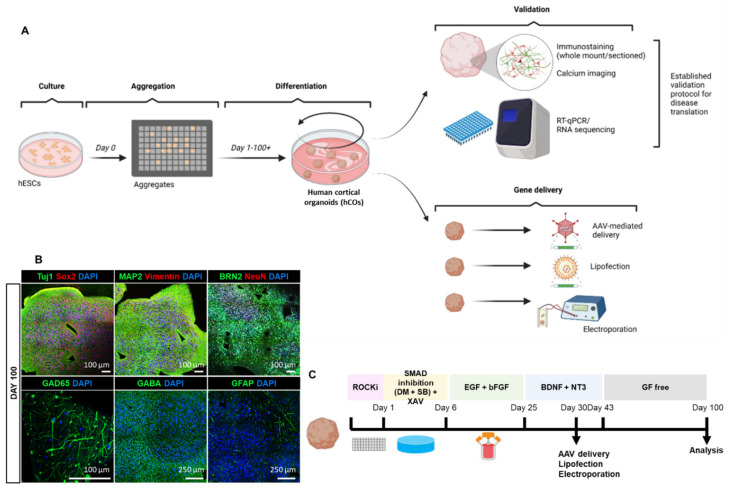
Establishment of hESC-derived cortical organoid. (**A**) Schematic illustration showing establishment of cortical organoid and gene delivery approaches. (**B**) Paraffin-sectioned immunohistochemical staining of cortical organoid at day 100 with antibodies for stemness (SOX2), neuronal (TUJ1, MAP2, NeuN), radial glial (VIM), cortical layer (BRN2), excitatory (GLUT1), and inhibitory neurons (GAD65, GABA) as well as astrocyte (GFAP). (**C**) Timeline of growth factor induction, gene delivery (i.e., Adeno-associated virus (AAV), lipofection, electroporation) and analysis of cortical organoid. Scale bars as indicated in the figure.

Before testing gene transfer into hCOs, we performed a detailed validation and characterization of the organoids including growth, differentiation, maturation to establish them as future human in vitro disease models through expression of human proteins associated with neurological conditions. To achieve this, we firstly initiated the generation of cortical organoids from hESCs by adapting an existing published protocol [1]. hESCs were maintained in culture prior to single-cell dissociation into an AggreWell^TM^ platform to enable high-throughput aggregation of highly reproducible organoids (Figure 1A). Following a protocol reported by Yoon and colleagues [1,38], we achieved neuro-ectodermal differentiation using the small molecule SB-431542 and dorsomorphin (a dual-SMAD inhibitor) followed by the Wnt-inhibitor, XAV-939. Thereafter, the neural medium was supplemented with epidermal growth factor (EGF) and basic fibroblast growth factor (bFGF) to enable neural stem cell proliferation and organisation [1]. Brain-derived neurotrophic factor (BDNF) and neurotrophin-3 (NT3) were further used for neuronal maturation. Subsequently, organoids were long-term maintained in growth factor-free neural medium (Figure 1C). The gene deliveries including viral transduction and non-viral trans transfection were performed (Figure 1C). The hCOs we generated recapitulate neural tube structure upon development and their proliferating population displayed volumetric growth over time (Supplementary Figure S1A). Starting with a size of 2 mm size at day 30, these cortical organoids expand in to approximately 10 mm in size at day 200 (Supplementary Figure S1B). In summary, we were able to culture hCOs in a robust and reproducible manner.

### 3.2. Homogenous Development of Diverse Cell Populations in hCOs

hCOs were analysed using whole-mount immunofluorescence staining at day 30 (Supplementary Figure S2A) and immunohistochemical staining of paraffin-embedded organoid sections at days 40, 50, and 100, respectively (Figure 1B, Supplementary Figure S2B,C). The antibodies used in this paper are listed in Supplementary Table S1. TUJ1 and MAP2-positive expression at day 30 demonstrates the presence of neuronal differentiation around the neural tube formations (Supplementary Figure S2). SOX2, a marker of progenitor cells, is present within the neural tube, confirming the proliferative state of these cells. Moreover, we observed high expression of the neuronal microtubule-associated protein tau at this stage of development (Supplementary Figure S2). To stain multiple proteins of interest from a single organoid, we established paraffin-embedding and serial sectioning of 4% PFA-fixed organoids from day 40 to 100 (Figure 1B, Supplementary Figure S2B,C). At day 40, we found expression of neuronal differentiation markers TUJ1, MAP2 and superficial cortical layer marker, BRN2, in organoids (Supplementary Figure S2B). At day 50, hCOs expressed the radial glial marker, vimentin (VIM), the neuronal maturation marker, NeuN, and post-synaptic marker, PSD95. Glutamatergic neuronal marker VGLUT1, GABAergic neuronal markers GAD65 and GABA, astrocyte marker GFAP and microglial markers IBA1 and CD68 were also present at day 50 (Supplementary Figure S2C). Furthermore, robust development of glutamatergic and GABAergic neuronal subtypes, and astrocytes was observed at day 100 (Figure 1B). Thus, we show here the presence of neuronal and glial cell types in our hCOs.

To assess the temporal expression of stem cell and maturation markers during organoid generation and maturation in more detail, we performed gene profiling by RT-qPCR across 4 different batches of hCOs at 7 different time-points (days 30, 40, 50, 60, 70, 100 to 200) (Supplementary Figure S3). The primer designs are listed in Supplementary Table S2. Each batch of hCOs and its culture media were prepared independently. OCT4 and SOX2, which are standard stem cell markers, exhibited similar transcriptional expression until day 70 with a slight increase at day 100 (Supplementary Figure S3). Neural progenitor cell (NPC) marker, PAX6, and radial glial cell marker Vimentin (VIM) showed increased expression in the early stages of organoid development (day 30 to 40). Neuronal markers TUJ1 and MAP2, were observed to transcriptionally increase at early stages of development (day 30 to 40) and subsequently maintained expression until day 70, and both markers at day 100 correlated with protein expression patterns captured in the immunostaining data (Figure 1B, Supplementary Figure S2). The forebrain markers FOXG1, SIX3, GSX2 and NKX2.1 showed consistent expression until day 70 with a marked increase at day 100 (Supplementary Figure S3). Cortical layer marker BCL11B (also known as CTIP2) gradually increased during hCO generation while microglial marker P2RY12, increased to peak expression at day 100. We next identified high gene expression of GRIN1 a glutamatergic neuronal marker, at day 100, consistent with neuronal subtype development in hCOs at late developmental stages (day 100). DLX5 and GABBR2, GABAergic neuronal markers, both gradually increased during organoid generation along with membrane proteins NKCC1 and KCC2, known to regulate GABA-ergic function [39,40], with increased expression at day 100, overall suggesting the development of GABA-ergic interneurons with hCOs development. Similar increases were also observed for astrocyte marker, GFAP and oligodendroglial marker OLIG2 by day 200 (Supplementary Figure S3). Overall, we demonstrate a reproducible neuronal and glia differentiation in hCOs under the outlined culture conditions.

### 3.3. Exploring Various Transfection Delivery Methods in hCOs

To explore the use of various other gene delivery methods and their applicability in hCOs, we tested Lipofectamine, electroporation and BrainFectIN^TM^ on 30-day old hCOs (Supplementary Figure S4). We used three different types of Lipofectamine (2000, Stem, and 3000) using a GFP expression plasmid (Supplementary Figure S4A). Lipofectamine 2000 and Lipofectamine Stem both exhibited a higher GFP signal than Lipofectamine 3000 at day 3 (Supplementary Figure S4A). Electroporation using the Neon Electroporation System from ThermoFisher was also tested with a mCherry expression plasmid at two different plasmid concentrations with 1µg of mCherry plasmid DNA resulting in higher expression than 0.4 µg (Supplementary Figure S4B). BrainFectIN, on the other hand, did not confer any mCherry expression (Supplementary Figure S4C). Here, we showed that Lipofectamine and electroporation are both efficient transfection approaches for gene delivery in hCOs. However, for stable and long-lasting gene expression for neurodegenerative disease model, we applied virus-mediated method on hCOs.

### 3.4. Adeno-Associated Virus-Mediated Gene Delivery in hCOs

**Figure 2:**
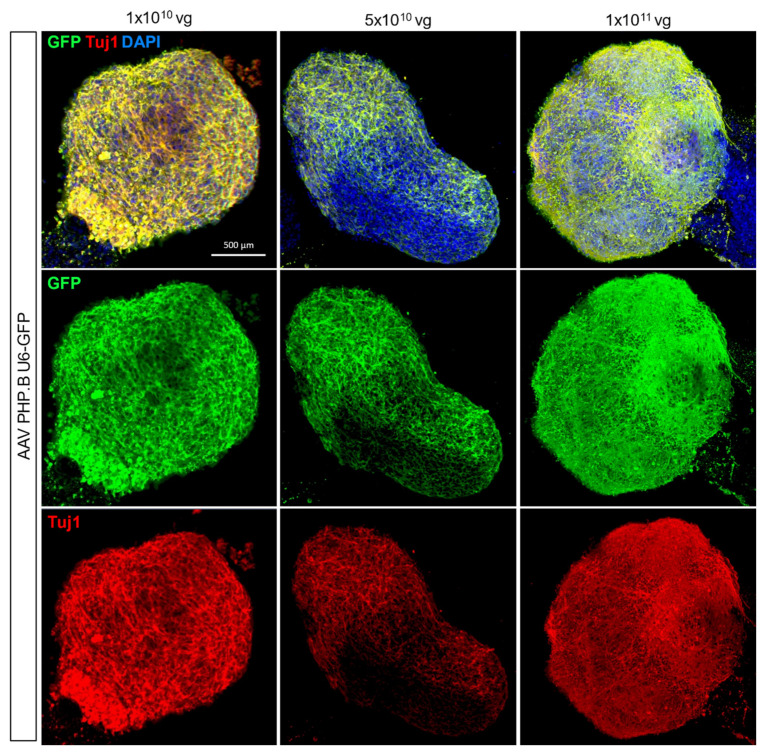
AAV-mediated gene delivery in human cortical organoids. 3 different titres (1 × 10^10^, 5 × 10^10^ and 1 × 10^11^ vg per organoid) of a AAV for ubiquitous GFP expression were tested and analysed by whole-mount immunostaining. GFP expression, neuronal marker (Tuj1) and nuclei (DAPI) were co-stained. Scale bar is 500 µm.

Adeno-associated virus-mediated gene delivery has become a method of choice for gene expression in vivo due to its widespread expression and ease of use in difficult-to-transduce tissues such as the brain [21]. Here we tested 3 different titres of a AAV subtype PHP.B for ubiquitous expression of the green fluorescence protein (GFP) in cells (1 × 10^10^, 5 × 10^10^ and 1 × 10^11^ vg) and found that 1 × 10^10^ vg optimally expressed GFP co-stained with the neuronal marker, TUJ1 3 days post-treatment (Figure 2). For neuron-specific gene delivery, we trialled 3 different AAV titres (5 × 10^11^, 5 × 10^12^ and 5 × 10^13^) for GFP expression under the pan-neuronal human Synapsin 1 promoter, AAV hSyn-EGFP, in 30-day-old hCOs (Supplementary Figure S5). We identified 5 × 10^12^ vg as the optimal concentration for AAV administration in hCOs while the highest concentration 5 × 10^13^ negatively impacted organoid viability, as disrupted organoid structure and morphology were observed (Supplementary Figure S5). A quantitative comparison of fluorescent GFP expression across the different methods further confirmed the optimal use of AAV as the approach of choice (Supplementary Figure S4D) with a representative image of GFP immunoblotting provided (Supplementary Figure S5D).

**Figure 3:**
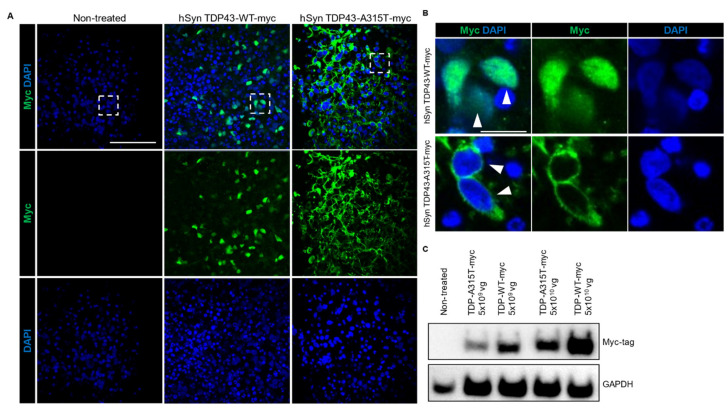
FTD/ALS human cortical organoid model. (**A**) AAV-mediated expression of TDP-43-WT and TDP-43-A315T in human cortical organoids showed widespread expression when immunostained with the c-terminal myc tag (green). DAPI stained the nucleus. Scale bar 100 µm. (**B**) Enlarged images of boxed area in (**A**) showed nuclear localisation of TDP-43 (myc-tagged; green; closed arrowheads) in TDP-43 WT cortical organoids while the TDP-43 in TDP-43 A315T organoids were localised to the cytoplasm (open arrowheads). Scale bar 20 µm. (**C**) Confirmation of AAV-mediated expression of TDP-43 in TDP-43-WT and TDP-43-A315T cortical organoids via western blotting with myc antibody. 2 different titers of AAV were used (5 × 10^9^ vg and 5 × 10^10^ vg). GAPDH is used as a loading control.

FTD and ALS are rapidly progressing fatal neurodegenerative diseases with similar disease aetiology and common neuropathological inclusions containing the nuclear TAR DNA-binding protein 43 (TDP-43), which is found in more than 90% of ALS and approximately 50% of FTD cases [28,41]. To establish a new model for studying these diseases in the future, we expressed non-mutant and mutant TDP-43 (with myc-tag) under hSyn promoter in hCOs using AAV and confirmed expression via immunostaining (Figure 3A) and western blotting (Figure 3B). In line with the human condition and transgenic TDP-43 mouse models of ALS/FTD, we found mis-localisation of TDP-43 in hCOs transduced with AAV hSyn TDP43-A315T-myc, while non-mutant TDP-43 expressed by AAV hSyn TDP43-myc showed normal nuclear distribution (Figure 3A) [30]. Notably, we observed the presence of disrupted organoid morphology in TDP-43 WT-expressing hCOs which were more pronounced and severe in the mutant TDP-43 A315T hCOs (Supplementary Figure S6). Herein, we showed that AAV can be successfully used to express genes in hCOs and expression of mutant TDP-43 can recapitulate key pathological features of FTD/ALS.

### 3.5. Neuron-Specific Calcium Functionality in hCOs

**Figure 4:**
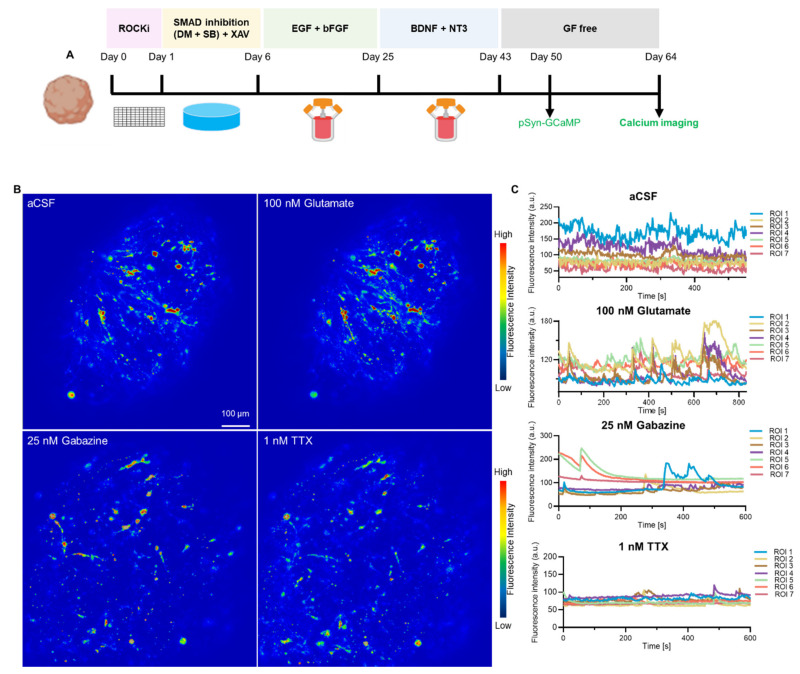
Normal neuronal network activity in human cortical organoid. (**A**) Protocol used to analyse AAV-mediated calcium indicator GCaMP expression under human Synapsin promoter. GCaMP AAV was added to cortical organoid at day 50 and measured after 14 days. (**B**) Heat maps of maximum fluorescence intensity of control aCSF, glutamate, gabazine, and TTX-treated cortical organoid. Scale bar is 100 µm. (**C**) Calcium traces of individual neurons were measured for each treatment (*n* = 7).

To verify the neuronal activity, we used an AAV to express calcium sensor protein jGCaMP7f under the control of the neuron-specific hSyn promoter in hCOs (Figure 4, Supplementary Figure S7). Fifty-day old organoids were transduced with AAV.hSyn-jGCaMP7f and analysed 2 weeks post-transduction (Figure 4A). Neurons in transduced hCOs exhibited spontaneous, asynchronous and transient calcium spikes, indicative of neuronal firing (Figure 4B,C). Addition of the neurotransmitter glutamate, an excitatory neuronal agonist, amplified peak amplitudes with synchronous neural activity, demonstrating increased calcium channel activation in hCOs while Gabazine, an GABA receptor antagonist, and Tetrodotoxin, a pan-sodium channel blocker decreased overall amplitude and frequency of calcium influx, in line with their inhibitive action on neuronal firing (Figure 4B,C, Supplementary Video S1). Our results demonstrate that AAV are suitable for delivering sensor proteins to determine the synaptic functionality of hCOs with the hCOs generated within our laboratory displaying the corresponding drug-induced modulation of neural activities (Figure 4).

## 4. Discussion

In this study, we generated hCOs using a previously published protocol [1,42] and demonstrated the potential of AAV for gene delivery. Across independently produced batches, hCOs showed reproducibility in all parameters measured including size, neuronal cell type markers, gene expression profile, and neuronal activity. This organoid model mimics the developmental process of the human brain and its cellular orientation with the formation of the neural tube and the presence of an api-co-basal axis. Both neuronal differentiation and synapse formation were present from day 30. Furthermore, we showed that the neurotropic AAV-PHP.B, which has widespread use for gene delivery in mouse models [21], is also suitable for efficient gene delivery in human organoids.

While protein/marker expression patterns in hCOs are most commonly visualized by whole-mount staining or cryosection staining [16,43], we adopted a histological protocol for paraffin embedding, sectioning and staining as typically performed for post mortem tissue analysis (e.g., mouse or human brains) [44]. This allowed co-staining of several markers in the same section and direct comparison of patterns across thin serial sections that captured the same cells at consecutive levels. Embedding several organoids in the same paraffin block further increases the throughput of this staining method. This method therefore allows assessment of a large variety of markers in several organoids at the same time.

Gene expression profiling of hCO over time demonstrated that stemness or progenitor markers were highly expressed at early-stage development during active proliferation, whereas neuronal differentiation and forebrain-related markers gradually increased in expression as hCOs aged. Accordingly, specific neuronal and non-neuronal cellular markers slowly increased as hCOs matured to day 100 (e.g., GRIN1, GABBR2, and P2RY12). The ability to culture functionally active brain organoids for extended periods of time, coupled with recent advances in single cell transcriptomics, has enabled the evolution of organoid differentiation protocols to identify various cell types, cell states and their developmental origins as the organoids age [14,45,46,47]. Hence, the use of hCOs and other brain organoid models is applicable not only to model and assess neurodevelopmental disorders but delve further into the realm of neurodegenerative diseases and other age-related studies.

In order to facilitate future functional studies and establishing new disease models to gain mechanistic insight into pathomechanisms, we tested common gene delivery tools used in vitro 2D cell culture studies and in vivo models. Amongst the non-viral gene transfer methods of polymer transfection, electroporation or BrainFectIN, we found lipofectamine 2000 and lipofectamine stem as the most effective tools for higher plasmid transfection with no overt cytotoxicity for organoids. We propose that studies requiring lower protein expression or less efficient transduction can make use of lipofectamine 3000 or an electroporation system, that allow for subtle gene expression levels. Although AAVs are frequently used in in vivo models and have entered clinical settings, to date, their utility for mediating gene delivery in human brain organoids remains underexplored. A recent study restricted expression to GABAergic medium spiny neurons in 3D human striatal spheroids, using an AAV expressing improved Cre (iCre) under a MiniPromoter for the striatal gene GPR88 (AAV-Ple94-iCre35). Use of AAV in this study enabled viral tracing and subsequent functional assays in intact and sliced assembloids to show that cortical neurons send axonal projections into striatal organoids and form synaptic connections [38]. Here, we show that AAVs can efficiently confer gene expression using both ubiquitous and neuron-specific promoters. We further demonstrate that AAV-mediated gene transfer does not compromise neuronal network function and indeed can be used to deliver sensor proteins to study neuron functionality in organoids. Finally, we demonstrate that expressing the human TDP-43 protein that has been associated with ALS and FTD recapitulates distinct neuropathological features of the human condition. The presence of active neuronal networks in hCOs further opens new avenues toward drug screenings for complex neurological disorders characterized by neural network dysfunction.

## 5. Conclusions

In this study, we successfully demonstrated the generation of hCOs based on previously published protocols and established that AAVs can be used as an effective gene delivery tool for rapid gene expression in hCOs. Expression of wild-type and mutant TDP-43 in hCOs under a neuron-specific promoter recapitulated cytoplasmic localisation of TDP-43, a pathological hallmark feature in FTD and ALS. In summary, the use of AAVs offers a quick method to express gene(s) of interest in human organoids and allows for in-depth analyses of physiological and pathological mechanisms underlying normal neuronal function and disease processes.

## Supplementary Materials

The following supporting information can be downloaded at: https://www.mdpi.com/article/10.3390/cells11203194/s1, Figure S1: Generation of human cortical organoid; Figure S2: Immunostaining of cortical organoid; Figure S3: Gene expression profile of human cortical organoids; Figure S4: Other gene delivery methods tested in human cortical organoids; Figure S5: Optimization of AAV titer on cortical organoid; Figure S6: Toxicity in hCOs treated with TDP-43 AAV;Video S1: Neuronal network activity in human cortical organoid; Table S1: Antibody for Immunocytochemistry; Table S2: Primer list for RT-1PCR.
