# Supplementary material for: Efficient Gene Expression in Human Stem Cell Derived-Cortical Organoids Using Adeno Associated Virus
Source: Cells. 2022 Oct 11;11(20):3194. doi: 10.3390/cells11203194 (PMC9601198; doi:10.3390/cells11203194)
Supplement: Supplementary file 1 [file cells-11-03194-s001.zip › cells-1944060-supplementary.pdf]

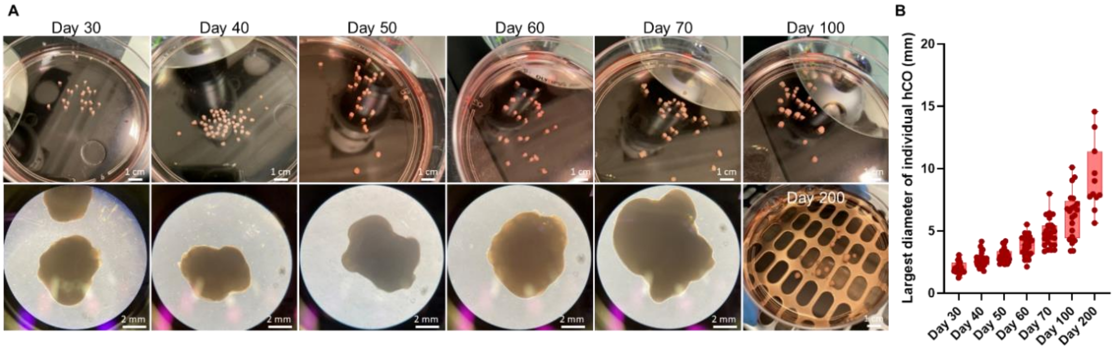

Figure S1. Generation of human cortical organoid. (**A**) Bright-field images of hCOs at day 30, 40, 50, 60, 70, and 100. Scale bar is 1cm (top panel) and 2 mm (bottom panel) (**B**) Quantification of diameter of cortical organoids at day 30, 40, 50, 60, 70, 100, and 200.

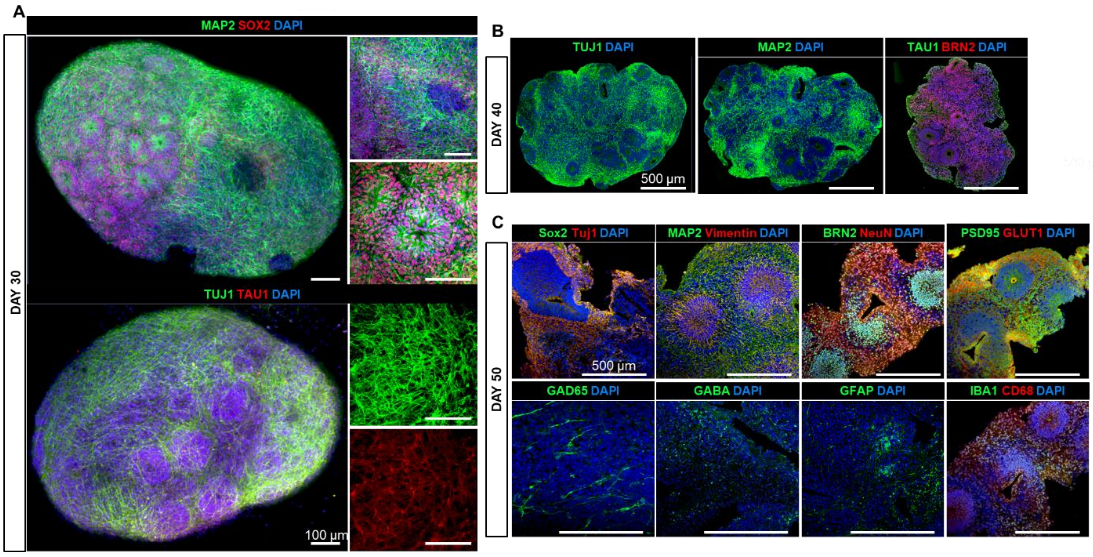

Figure S2. Immunostaining of cortical organoid. (**A**) Whole mount staining of hCOs at day 30. Stemness marker (SOX2), microtubule marker (TAU1) and neuronal markers (TUJ1, MAP2) were expressed. Scale bar is 100 µm. (**B**) Paraffin sectioned immunohistochemical staining of hCOs at day 40. The neuronal (TUJ1, MAP2), microtubule (TAU1) and cortical layer (BRN2) markers were present at this stage. Scale bar is 500 µm. (**C**) Paraffin sectioned immunohistochemical staining of hCOs at day 50. The stemness (SOX2), neuronal (TUJ1, MAP2, NeuN), radial glial (VIM), cortical layer (BRN2), post-synaptic (PSD95), excitatory (GLUT1) and inhibitory neurons (GAD65, GABA), astrocyte (GFAP) and microglial (IBA1, CD68) markers were present at this stage. Scale bar is 500 µm.

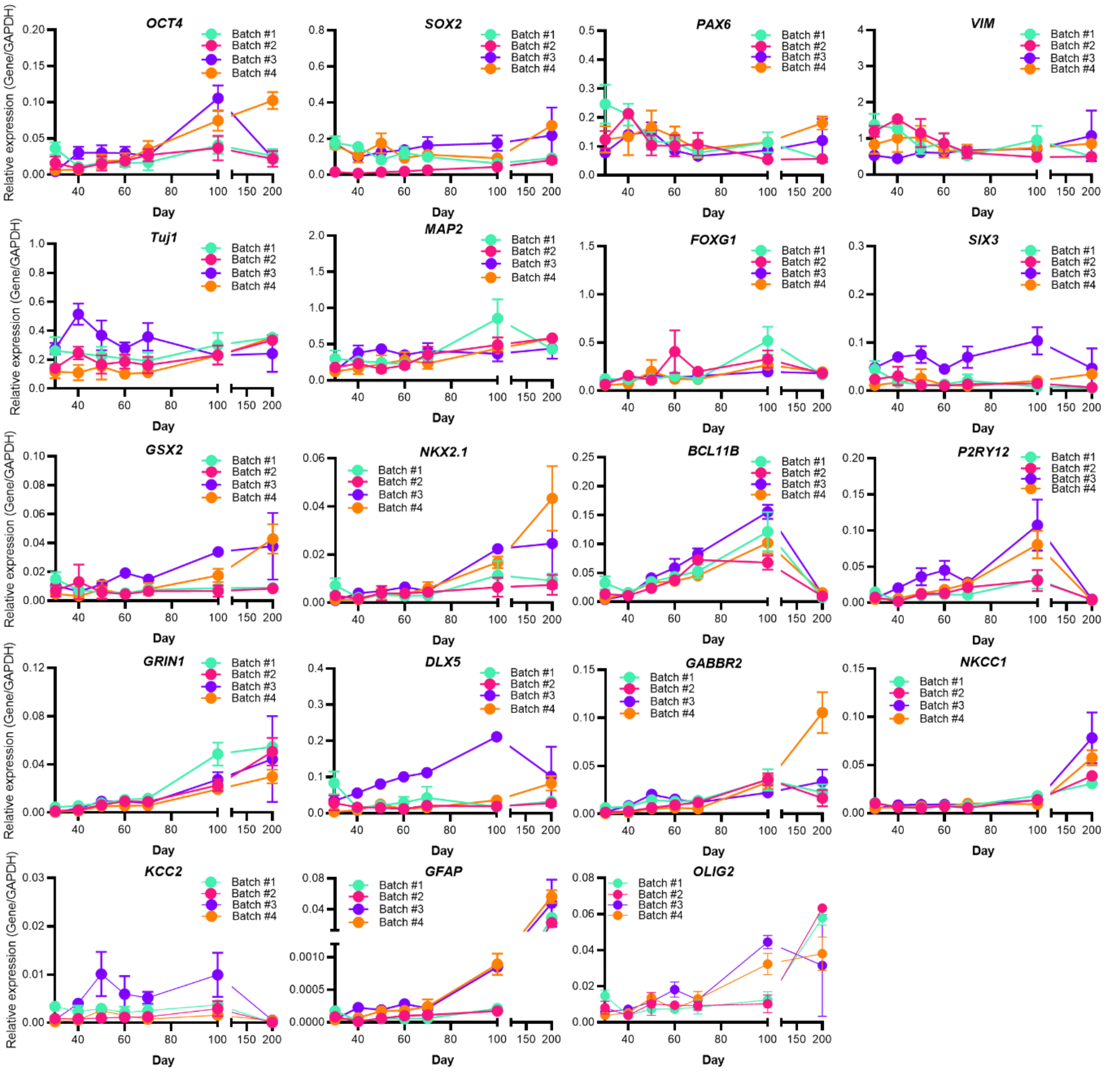

Figure S3. Gene expression profile of human cortical organoids. The qRT-PCR data of day 30, 40, 50, 60, 70, 100, and 200 day-old cortical organoid. To show reliable production of cortical organoid, 4 separate batches of cortical organoids are used for analysis. Gene expression profile showed relative expression of OCT4, SOX2, PAX6, VIM, TUJ1, MAP2, FOXG1, SIX3, GSX2, NKX2.1, BCL11B, P2RY12, GRIN1, DLX5, GABBR2, NKCC1, KCC2, GFAP, and OLIG2 compared to house-keeping gene (GAPDH).

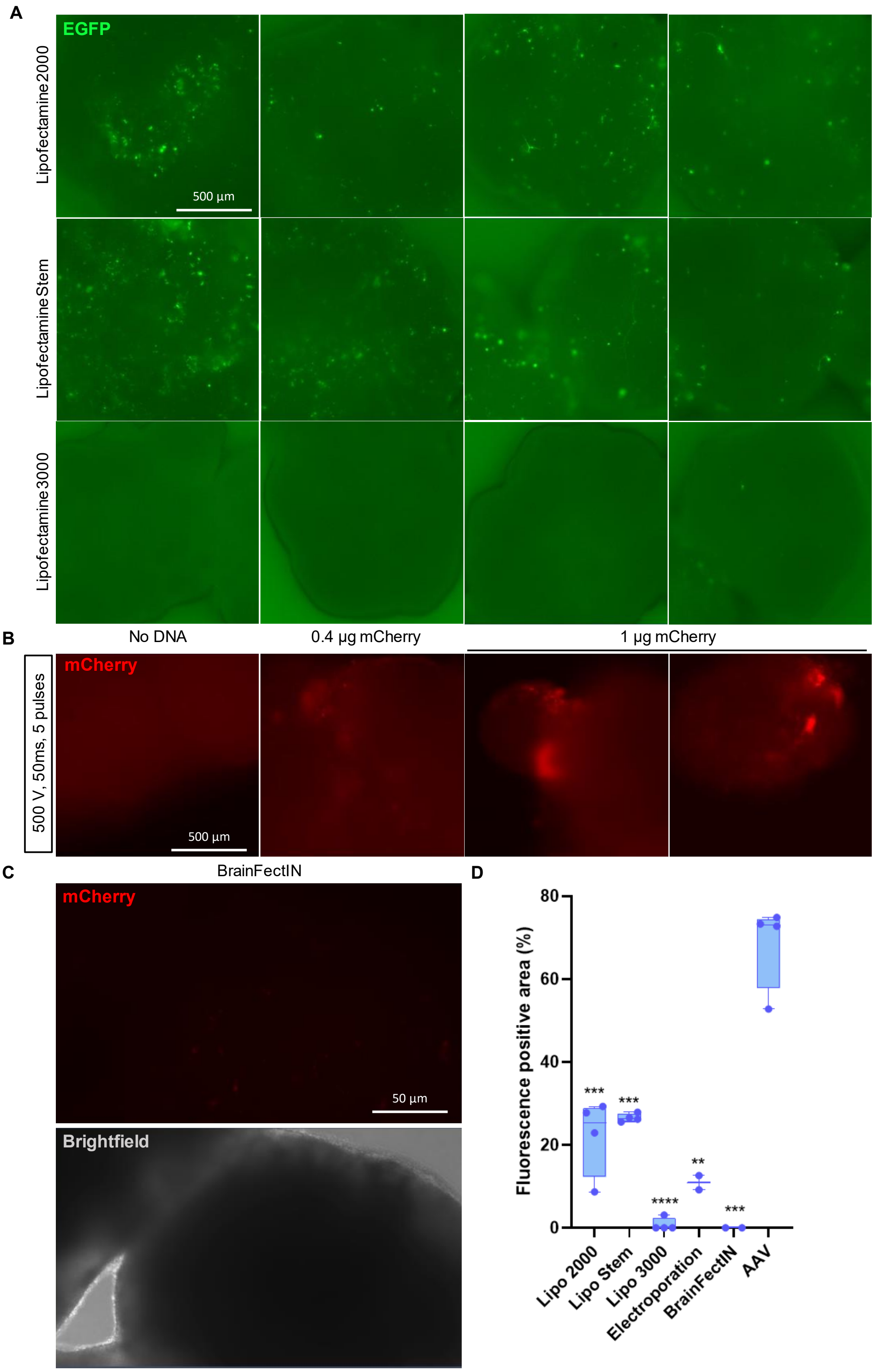

Figure S4. Other gene delivery methods tested in human cortical organoids. (**A**) Different lipofectamine (2000, Stem and 3000) were tested on day 30-old hCOs to express GFP. Scale bar is 500 µm. (**B**) Electroporation of a mCherry-expressing plasmid at different concentration (control, i.e., no DNA, 0.4 µg and 1 µg). Scale bar is 500 µm. (**C**) Transfection with BrainFectIN reagent of a mCherry-expressing plasmid. Scale bar is 50 µm. (**D**) Quantification of fluorescent-positive area per organoid in different gene delivery methods

including lipofectamine (2000, stem and 3000), electroporation and BrainFectIN (** $p < 0.005$, *** $p < 0.001$ vs. AAV and **** $p < 0.0001$ vs. AAV).

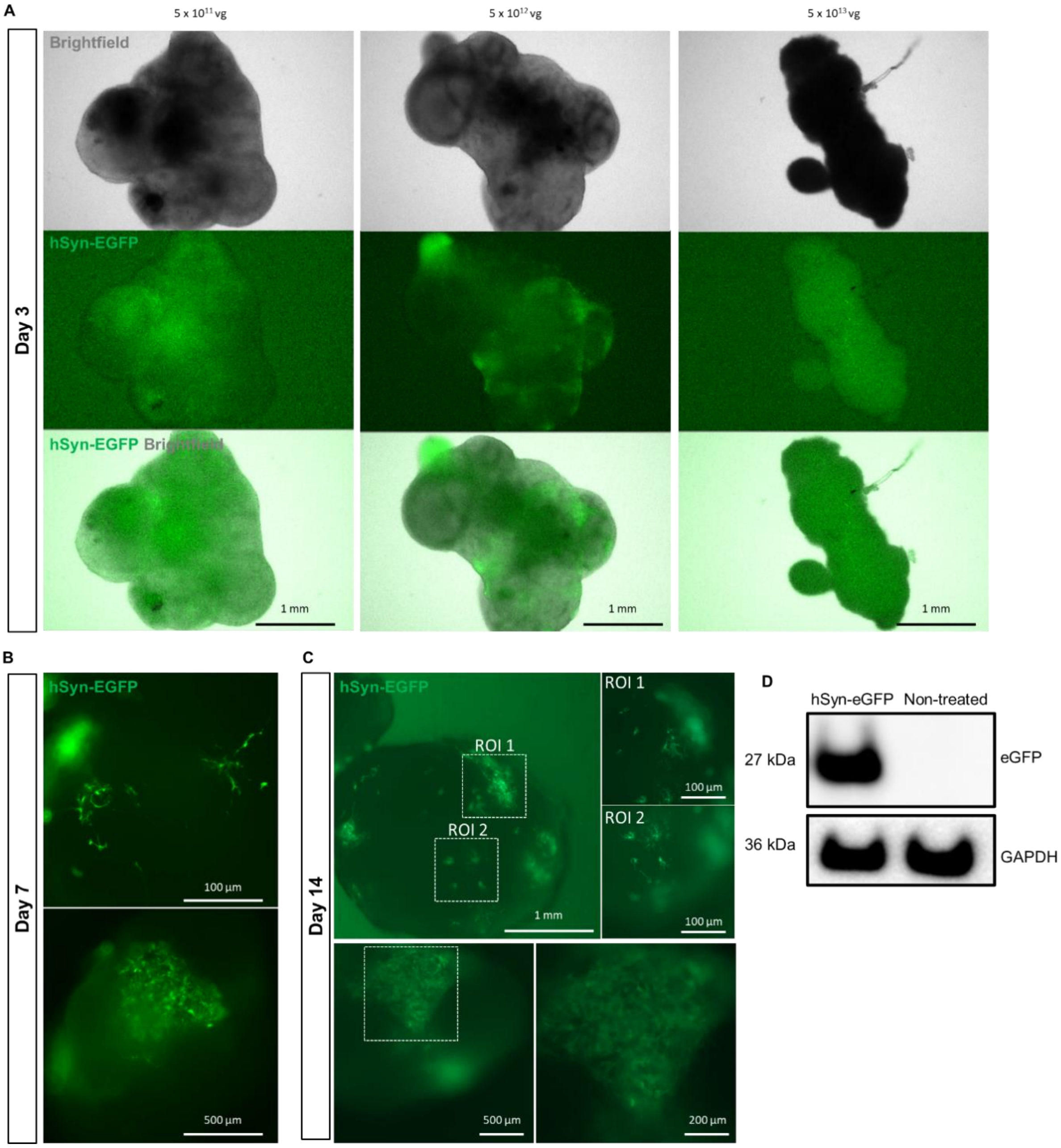

Figure S5. Optimization of AAV titer on cortical organoid. (**A**) 5×10^{11}, 5×10^{12} and 5×10^{13} vg of AAV pSyn-EGFP added to day 30-old cortical organoids and fluorescence expression was analysed 3 days thereafter. Scale bar is 1 mm. Further analysis of 5×10^{12} vg AAV-treated cortical organoid (**B**) at day 7 and (**C**) day 14 were performed. Scale bars as shown in figure. (**D**) Western blot analysis of AAV-treated cortical organoid using optimized AAV titre with EGFP antibody (anti-eGFP, GAPDH for housekeeping).

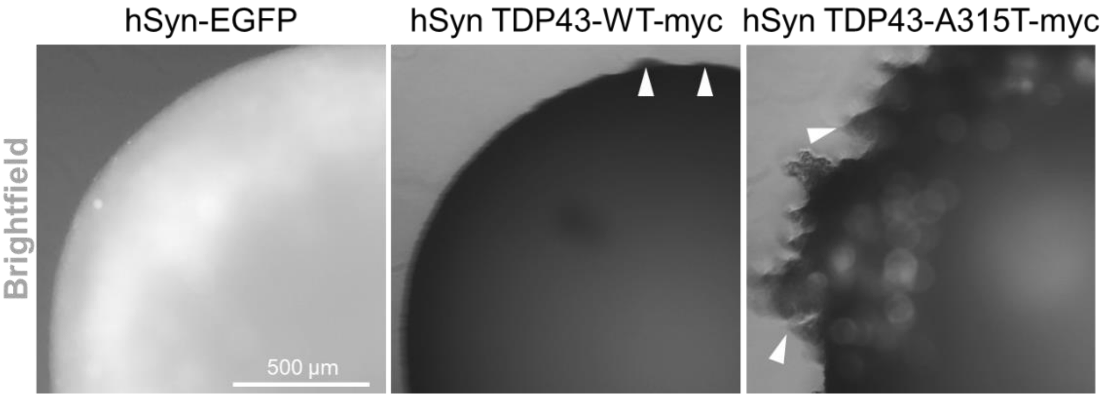

Figure S6. Toxicity in hCOs treated with TDP-43 AAV. The hCOs were treated with hSyn-EGFP, hSyn TDP43-WT-myc and hSyn TDP43-A315T-myc AAVs displayed morphological disruption (arrowheads).

Video S1. Neuronal network activity in human cortical organoid. Videos of AAV-mediated expression of the calcium indicator jGCaMP7 under the human synapsin promoter for control (aCSF), glutamate, gabazine and TTX treated cortical organoids. Videos have been uploaded to Zenodo (https://doi.org/10.5281/zenodo.7080334).

Table S1. Antibody for Immunocytochemistry.

**Type**	**Host**	**Supplier**	**Catalog #**	**Dilution Ratio**
TUJ1	Mouse	Sigma-Aldrich	T8578	1:200
TUJ1	Rabbit	Covance	PRB-435P	1:500
TAU1	Mouse	Chemicon	MAB3420	1:500
SOX2	Rabbit	Chemicon	AB5603	1:200
MAP2	Chicken	Abcam	ab5392	1:500
BRN2	Rabbit	Abcam	Ab94977	1:500
Vimentin	Mouse	Proteintech Group	65039	1:200
NeuN	Mouse	Chemicon	MAB377	1:500
PSD95	Rat	Chemicon	MAB1596	1:500
GLUT1	Rabbit	Chemicon	07-1401	1:500
GAD65	Mouse	Abcam	Ab26113	1:500
GABA	Rabbit	Sigma-Aldrich	A2052	1:500
GFAP	Mouse	Sigma-Aldrich	G3893	1:500
IBA1	Goat	Abcam	Ab5076	1:500
CD68	Rat	Bio-Rad	MCA19957T	1:100
Myc	Rabbit	Abcam	Ab9106	1:500

Table S2. Primer list for qRT-PCR.

**Type**	**F Primer**	**R Primer**
OCT4	CAGTGCCCGAAACCCACAC	GGAGACCCAGCAGCCTCAAA
SOX2	TTCACATGTCCCAGCACTACCAGA	TCACATGTGTGAGAGGGGCAGTGTGC
PAX6	TGGGCAGGTATTACGAGACTG	ACTCCCGCTTATACTGGGCTA
VIM	CTCCGGGAGAAATTGCAGGA	TTCAAGGTCAA GACGTGCCA
Tuj1	GGCCTTTGGACATCTCTTC	CTCCGTGTAGTGACCCTTG
MAP	GAGAATGGGATCAACGGAGA	CTGCTACAGCCTCAGCAGTG
FOXG1	AACCTGTGTTGCGCAAATGC	AAACACGGGCATATGACCAC
SIX3	AGCAGAAGACGCATTGCTTC	ACCAGTTGCCTACTTGTGTG
GSX2	ATGTCGCGCTCCTTCTATGTC	ATGCCAAGCGGGATGAAGAAA
NKX2.1	AGCACACGACTCCGTTCTC	GCCCACTTTCTTGTAGCTTTCC

NKCC1	TAAAGGAGTCGTGAAGTTTGGC	CTTGACCCACAATCCATGACA
KCC2	AGGAAAGCAGTCCCTTCATCA	GCCTCTTCATGCTCCCTACTT
GRIN1	CGTGAGTCCAAGGCAGAGAA	TCTTTCGCCTCCATCAGCAG
DLX5	TTCCAAGCTCCGTTCCAGAC	GAATCGGTAGCTGAAGACTCG
P2RY12	TTTGTGTGTCAAGTTACCTCCG	CTGGTGGTCTTCTGGTAGCG
BCL11B	GGTGCCTGCTATGACAAGG	GGCTCGGACACTTTCCTGAG
GFAP	GAGAACCGGATCACCATTCC	CCCAGTCTGGAGCAACCTAC
OLIG2	AGGACAAGAAGCAAATGACAG	TCCATGGCGATGTTGAGG
GABBR	ACCAACTTCTTCGGGGTCAC	CACCTCCCTGCTGTCTTGAA
GAPDH	CATGAGAAGTATGACAACAGCCT	AGTCCTTCCACGATACCAAAGT
